# Adult brain T1 and T2 values measured at 3 T using magnetic resonance fingerprinting with phantom validation

**DOI:** 10.1002/acm2.70289

**Published:** 2025-12-15

**Authors:** Kalina V. Jordanova, Stephen E. Ogier, Stephen E. Russek, Cassandra M. Stoffer, Karl F. Stupic, Guido Buonincontri, Mathias Nittka, Kathryn E. Keenan

**Affiliations:** ^1^ National Institute of Standards and Technology Boulder Colorado USA; ^2^ Magnetic Resonance Siemens Healthineers AG Erlangen Germany

**Keywords:** T1, T2, phantom validation, magnetic resonance fingerprinting

## Abstract

**Background:**

For clinical implementation of quantitative MRI methods, it is necessary to understand the expected range of nominally healthy tissue properties. Additionally, to compare measurements across MRI systems or provide guidance for clinically meaningful change, these nominally healthy measurements should be completed with phantom validation of the measurement system and method.

**Purpose:**

To measure T1 and T2 relaxation times on 20 participants using magnetic resonance fingerprinting (MRF) concurrently with phantom measurements for validation.

**Methods:**

T1 and T2 relaxation times were measured at 3 T using MRF on 20 participants. To assess longitudinal reproducibility, a phantom was scanned in the same session as each in vivo measurement, which resulted in 11 phantom measurements over the 99‐day study duration. At each imaging session, phantom and in vivo test‐retest measurements were made. Phantom test‐retest repeatability was evaluated along with longitudinal reproducibility. Additionally, in vivo test‐retest repeatability was evaluated for brain regions of various sizes, and mean in vivo measurements for cerebrospinal fluid (CSF), gray matter (GM), and white matter (WM) are presented.

**Results:**

Over the study duration, phantom measurements were repeatable and reproducible, and no changes were detected in the MRF measurement of T1 and T2 relaxation times on this system. For in vivo measurements, test‐retest variation for CSF, GM, and WM was 2.3%, 0.31%, 0.57% for T1 and 8.3%, 1.9%, 1.3% for T2. We observed that the test‐retest variation of T1 and T2 measurements increased as the segmented volume fell below 1 cm^3^. Mean and standard deviation of T1 and T2 were calculated over all participants for CSF, GM, and WM.

**Conclusions:**

Phantom measurements characterized the measurement accuracy, repeatability, and reproducibility throughout the study. The MRF T1 and T2 measurements of normal brain tissues at 3 T are validated through concurrent phantom measurements, and mean values were reported for each tissue. This study provides one data point to answer the question of how often phantom quality assessment must be done in tandem with in vivo measurements.

## 1 INTRODUCTION

Clinical adoption of quantitative relaxometry requires an understanding of nominally healthy values.^1^ Without an understanding of the expected range of healthy values, it is not possible to distinguish disease states using quantitative relaxometry. Previous studies have measured nominally healthy tissue relaxation;^2^, ^3^ however, the methods used vary, and many studies have fewer than 10 participants. Additionally, in much of the previous work, the authors do not report the accuracy of the measurement method using a standardized phantom.

Standardized reference objects, or phantoms, can be used to assess MRI protocol accuracy. Studies have shown the value of phantom use following scanner upgrades^4^, ^5^ and when translating quantitative MRI methods to the clinic.^6^ A phantom can be used to establish the test‐retest repeatability, the reproducibility of a measurement method over time, and the accuracy of a measurement. In this case, we define accuracy to be the deviation from a reference measurement. This is especially valuable when trying to determine expected values for quantitative parameters (e.g., for nominally healthy tissue or to define a disease threshold).

Magnetic resonance fingerprinting (MRF) is an efficient and repeatable method to measure tissue T1 and T2,^7^ and previous studies have established the reproducibility of the method over 1 month using a phantom^8^ and the repeatability of the method in vivo.^3^, ^7^, ^9^ Although some in vivo studies have used a phantom,^10^ the phantom measurements have been limited to single time‐points and did not continue throughout the study duration. There have not been MRF in vivo measurements that use a standardized reference object throughout the study to characterize the measurement accuracy, repeatability, and reproducibility. It has not been established whether a phantom measurement at a single time‐point is sufficient to ensure measurement validity, or whether the continued use of a reference object is necessary to ensure that the MRI system is operating within specification for the duration of the study, and the resulting measurements are valid.

Given the repeatability and reproducibility of MRF, as shown by previous studies, we used MRF to measure T1 and T2 relaxation times on 20 participants and concurrently on a standardized phantom over approximately 3 months. The phantom measurements serve two purposes: first, to establish the measurement accuracy of this MRF implementation, and second, to verify the repeatability and reproducibility of the measurement system throughout the acquisition of the in vivo data. The phantom validation results allow us to report mean brain T1 and T2 relaxation times over 20 adult participants with an understanding of the method's measurement accuracy.

## 2 METHODS

### 2.1 Overview

T1 and T2 relaxation times were measured using MRF on 20 participants, and a phantom was scanned, either immediately before or after each in vivo measurement. The phantom scanning was accomplished between the adjacent sessions of two participants, such that phantom imaging sessions were concurrent with the in vivo measurements. The study duration was 99‐days and included 11 phantom imaging sessions. The participant and phantom imaging sessions were scheduled based on the availability of the participants and the MRI system; these were not at regular intervals. At each imaging session, phantom and in vivo test‐retest measurements were made. Phantom measurement accuracy was evaluated along with test‐retest repeatability and longitudinal reproducibility. Additionally, in vivo test‐retest repeatability was evaluated, and mean in vivo measurements for cerebrospinal fluid (CSF), gray matter (GM), and white matter (WM) are presented.

### 2.2 MRF sequence

All scans were conducted on a 3 T Siemens scanner (MAGNETOM Prisma fit, Siemens Healthineers AG, Forchheim, Germany) using the body transmit coil and a 20‐channel receive head/neck coil. MRF data were acquired using a 2D Fast Imaging with Steady State Precession (FISP) spiral acquisition research sequence^11^ with 1500 measurements, 2 ms echo time, 12.1 ms to 15.0 ms repetition times, and 0° to 74° flip angles with constant RF phase. The spiral interleave order was 82.5° increment between readouts.^12^ The FISP echotrain is preceded by an adiabatic, non‐selective inversion pulse with a 21 ms inversion time. For detailed acquisition parameters, including the temporal variation pattern for the repetition times and flip angles, please refer to Yokota et al.^13^ Seven slices were acquired with a scan time of 20 s each. The reconstructed in‐plane resolution was 1 mm by 1 mm with 5 mm slice thickness, with a field of view of 256 × 256. The 2D images were reconstructed for each spiral readout, and the k‐space trajectories were corrected for system imperfections (e.g., eddy currents).^14^ The duration of the spiral readout is 6 ms, which is sufficiently short that blurring is not observed in the brain. Therefore, no dedicated deblurring is applied to compensate for any off‐resonances. A straight‐forward NUFFT was applied to reconstruct the single spiral readouts. Individual coil channels were combined after the spiral reconstruction using an adaptive coil combine algorithm designed to preserve optimum signal‐to‐noise. Then the signal‐course of 1500 time points in each image pixel is SVD compressed to 50 points to accelerate the reconstruction time and reduce the computer memory requirements. The sequence and dictionary used in this study are the same as the FDA‐approved implementation from this vendor.

The dictionary range was 10–4500 ms for T1 and 2–3000 ms for T2, with increasing step sizes as T1 or T2 increased.^13^ Specifically, the step sizes over each range of T1 were 10 ms (for T1 10–100 ms), 20 ms (100–1000 ms), 40 ms (1000–2000 ms), and 100 ms (2000–4500 ms), and the step sizes for T2 were 2 ms (2–100 ms), 5 ms (100–150 ms), 10 ms (150–300 ms), 50 ms (300–800 ms), 100 ms (800–1600 ms), and 200 ms (1600–3000 ms). The dictionary has an additional dimension containing simulated signal evolutions with scaled flip angles to consider regions with nonideal transmit field (B1). Specifically, it covers relative B1 values from 0.6 to 1.4 (step size 0.01), where a value of 1.0 corresponds to the ideal nominal transmit field. However, the matching is not stable over three dimensions (T1, T2, and B1); as a result, the B1 value for each pixel is obtained from a measured B1 map^15^ before the matching process in the T1 and T2 dimensions. Dictionary matching is accomplished by determining the closest inner product match to the signal time course in each pixel, and an approximate nearest neighbor search is used to accelerate the matching process.^16^ For shorter reconstruction times and reduced memory requirements, the dictionary is compressed to 50 main components in the time domain using a singular value decomposition (SVD) approach.^17^

The B1 mapping method is described by Chung et al.^15^ As described by the authors, the B1 mapping method is not sufficiently accurate for T1 < 400 ms. Given this constraint, voxels with a lower T1 relaxation time were masked in the quantitative images. For T1 < 400 ms, the accuracy of the B1 mapping method can be increased by using a longer TR. However, for the application to brain imaging, it is appropriate to keep the B1 mapping acquisition short and focus on T1 > 400 ms. Initial verification of the MRF sequence was completed using an ISMRM/NIST system phantom (results not shown).

### 2.3 Phantom study

**FIGURE 1:**
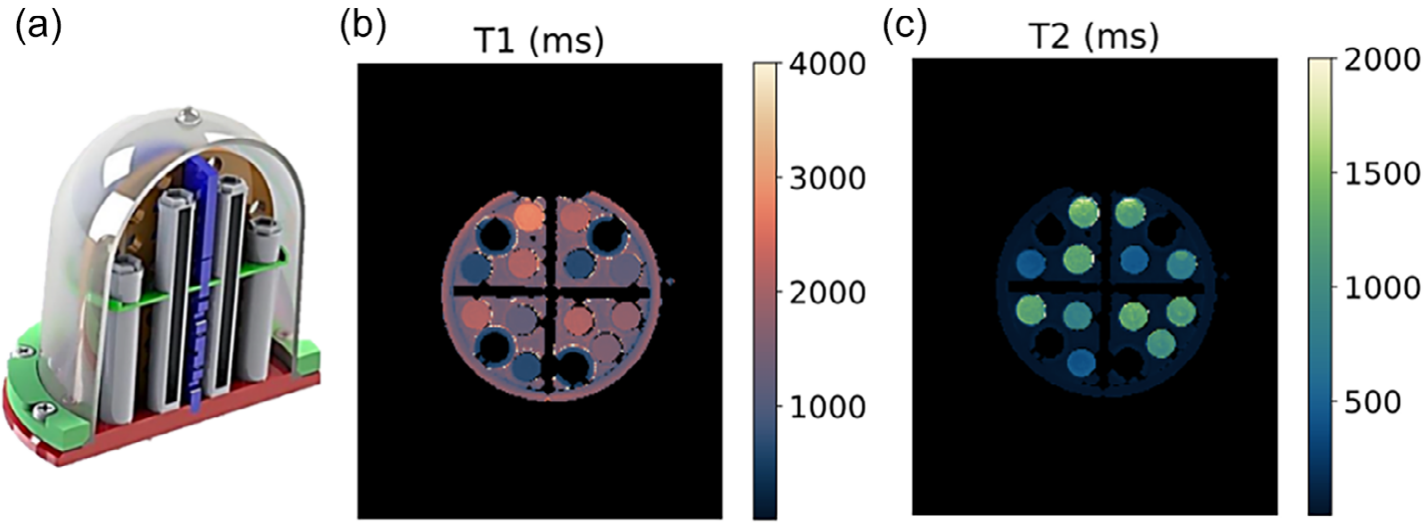
(a) Rendering of the phantom used in the study, from the phantom manufacturer's manual. Example MRF T1 (b) and T2 (c) maps for the phantom. Four fat mimics are masked in the images due to T1 < 400 ms. The phantom was imaged in the same coil used for in vivo imaging, held in a 3D‐printed holder for repeatable positioning. The holder positioned the phantom with the material tubes along the B0 direction of the MRI system, with the phantom base plate covering the opening of the head coil. The phantom was stored in the scan room between imaging sessions to minimize temperature variation.

This study was part of a larger study that also acquired quantitative diffusion measurements in the same set of participants. As a result, the phantom used in the study needed to simultaneously represent the T1 and T2 relaxation properties as well as diffusion properties. Here we consider only the T1 and T2 properties of the phantom. A commercially available breast phantom^18^ (CaliberMRI, Boulder, CO, USA) (Figure 1) was modified for this study: the diffusion portion of the phantom was removed from the backboard and used for scanning. The phantom acquisitions used the same coils, sequence, and reconstruction as the in vivo acquisition. A 3D‐printed holder was used to ensure consistent positioning of the phantom in the coil throughout the study. The holder positioned the phantom with the sample vials along the B0 direction of the MRI system, with the phantom base plate covering the opening of the head coil. The phantom contains aqueous solutions of polyvinylpyrrolidone (PVP), with 0 %, 10 %, 25 %, and 40 % w/w PVP concentrations. Some solutions have repeated vials in the phantom. Additionally, there are vials that contain a fat mimic material, and a fibroglandular (FBG) mimic material is the background fill of the phantom.

Phantom regions of interest (ROIs) were identified by selecting a central, circular region with a diameter of 8 mm in each vial over six slices. (Vials are approximately 16 mm in diameter.) ROIs for FBG were found by selecting four regions of the background fill of the same size as the vial ROIs, albeit with all four regions in the single slice that was clear of the tubes (Figure S1).

The MRI measurements were compared to NMR‐reference measurements to examine measurement accuracy. Reference NMR data were collected following the standard NIST protocol for measuring relaxation times described in NIST special publication SP‐250‐97,^19^ with one modification: the T2 measurements were made using spin echo rather than Carr‐Purcell‐Meiboom‐Gill (CPMG) methods. An aliquot of each phantom material was measured using a variable field, variable temperature NMR system (Tecmag Redstone HF‐1 NMR spectrometer paired with Tecmag TNMR software), where data was collected at 3 T and sample temperature was controlled using a localized sample thermometer as described in the SP‐250‐97 (sections 4 and 5). T1 was measured using an inversion recovery sequence with 20 logarithmically‐spaced TI times at 20°C.^19^ The T2 measurements were made at 20°C with single echo spin echo methods using 51 echo times logarithmically spaced from 2.026 to 2502 ms. T_2_ was calculated by fitting a decaying exponential to the integrated echo intensity. The NMR reference values at 20°C are reported in Table 1. To examine measurement accuracy, the percent deviation of the MRF T1 and T2 measurements from the NMR‐measured value is calculated.

**TABLE 1:** Reference T1 and T2 relaxation times for the phantom materials measured using NMR.

Material	T1 (ms)	T2 (ms)
0 % PVP	2872 (48)	1217 (60)
10 % PVP	2151 (32)	1245 (27)
25 % PVP	1304 (20)	962 (17)
40 % PVP	656 (10)	472 (10)
FBG	1321 (15)	41 (2)

To assess MRF measured T1 and T2 repeatability, test‐retest measurements were made. The phantom was not removed from the bore; the test‐retest measurements are simply a repeat of the acquisition. Phantom test‐retest accuracy was visually assessed using Bland‐Altman plots. Test‐retest variation was calculated for each material and relaxation parameter as

(1) $$Var=\frac{1}{N}\;\sum_{i=1}^{N}\frac{\left|test_{i}-retest_{i}\right|}{\left(test_{i}+retest_{i}\right)/2}*100$$

where N is the number of measurements and $test_{i}$ and $retest_{i}$ are the ROI mean test‐retest measurements. To examine measurement reproducibility over time, the coefficient of variation (CV) was calculated for the MRF T1 and T2 measurements for each material over the 99‐day study.

The ambient temperature of the scanner room was recorded at each imaging session. The phantom was stored in the scanner room to minimize thermal variations.

### 2.4 In vivo study

The MRF sequence was used to acquire repeated T1 and T2 measurements on 20 healthy participants (7 female, 13 male, ages 22–68 years, mean age 44.05 years) in accordance with Institutional Review Board (IRB) guidelines. The Advarra IRB reviewed and approved this study on behalf of the National Institute of Standards and Technology. All subjects provided informed consent.

Volumetric, 3D T1‐weighted images were acquired for brain region segmentation. The 3D inversion recovery T1‐weighted sequence had 0.8 mm isotropic resolution, 2400 ms repetition time, 1000 ms inversion time, and 2.1 ms echo time. Brain region segmentation was performed using SynthSeg^20^, ^21^ (v2.0) on the T1‐weighted image to segment WM (cerebral WM), GM (cerebral cortex), and CSF (lateral ventricles). Smaller brain regions were also obtained from SynthSeg for further analysis. Segmentations were transformed to the MRF image space using the scanner's affine matrices with the *processing* module of Python's NiBabel package.

Participant test‐retest accuracy was assessed using Bland‐Altman plots for each brain region. Additional test‐retest accuracy and Bland‐Altman plots were created for smaller brain regions, as segmented by the SynthSeg output. Histograms of the repeated measurements for each participant and region were visually compared. Test‐retest variation was calculated for each tissue and relaxation parameter using Equation 1 with N as the number of participants.

Mean and standard deviation were found for each brain region, participant, and relaxation parameter, and a linear fit was made of the means as a function of age to examine age‐related trends. Fit quality was evaluated using p and R^2^ values. Overall mean T1 and T2 relaxation times were reported for each brain region.

Finally, all T1 and T2 relaxation time maps use the color map scheme specified by Fuderer et al.^22^

## 3 RESULTS

### 3.1 Phantom measurements

**FIGURE 2:**
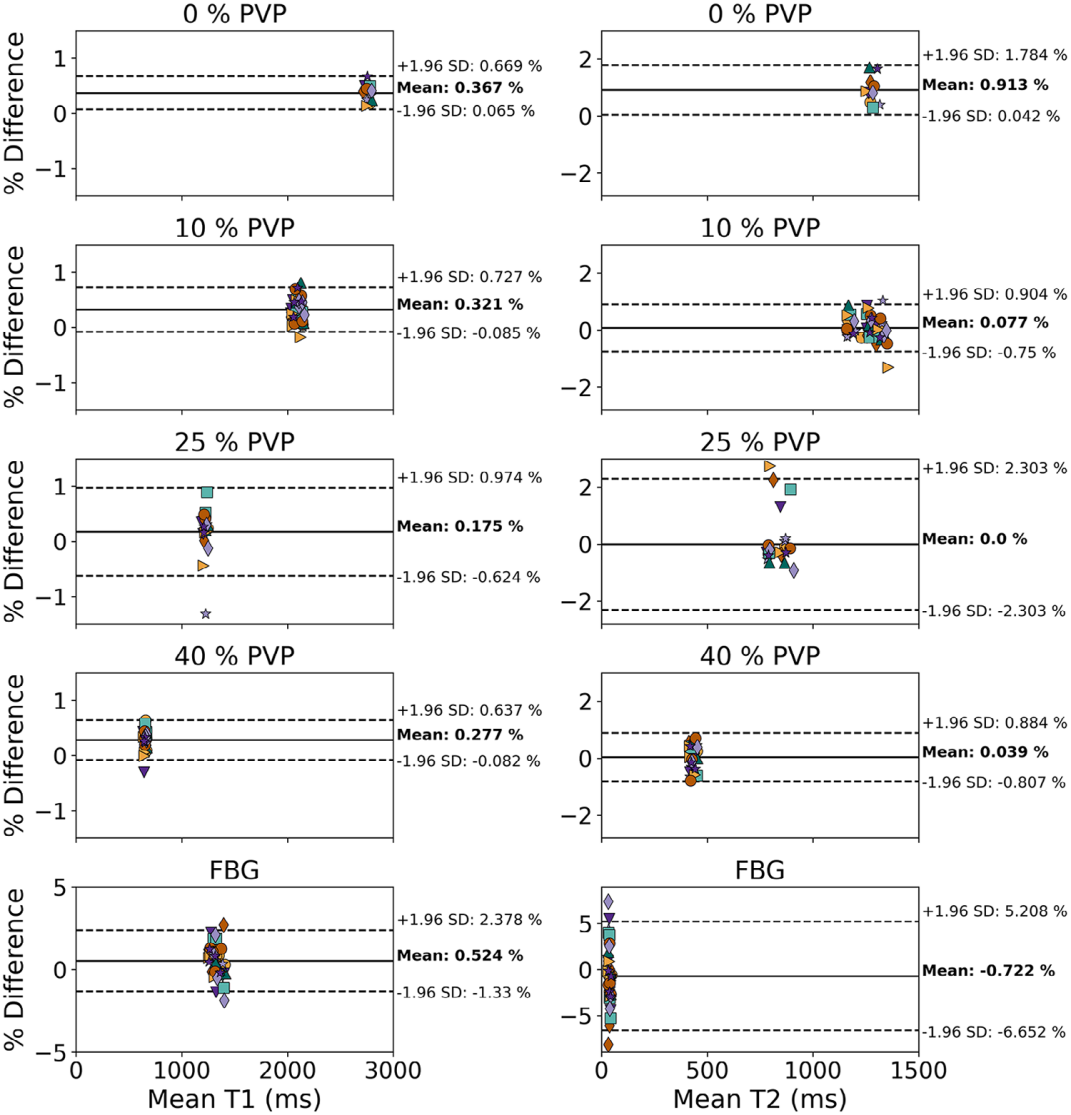
Bland–Altman plots of T1 and T2 for each phantom sample test‐retest measurement. Each marker represents one of the 11 measurement dates. The phantom contains multiple samples of all materials except 0 % PVP, which results in more than 11 points per plot (except for 0 % PVP). Percent differences between each test‐retest measurement are calculated and plotted as a function of the mean test‐retest value. The mean (solid line) and 1.96*standard deviation (dashed lines) were calculated for each measurement type. PVP, polyvinylpyrrolidone.

Example MRF T1 and T2 maps for the phantom are shown in Figure 1. We note that the four fat mimics are masked in the images due to T1 values less than 400 ms, and as a result, the fat samples are excluded from the analysis. Bland‐Altman plots for each ROI in the test‐retest measurements are shown (Figure 2). There is minimal bias between the test‐retest measurements across all materials; T1 and T2 mean differences are < ± 1.0 %. The FBG T2 measurements had the greatest spread with the 95% CI from +5.2 % to −6.7 %. Test‐retest variation calculated using Equation (1) was below 1 % for all measurements except FBG T2, which had 2.44 % variation (Table 2).

**TABLE 2:** T1 and T2 test‐retest variation for each phantom material over the 99‐day study shows a test‐retest variation of less than 1 % for all measurements except FBG T2, for which the variation was 2.44 %.

Material	T1 test‐retest variation (%)	T2 test‐retest variation (%)
0 % PVP	0.37	0.91
10 % PVP	0.33	0.32
25 % PVP	0.35	0.78
40 % PVP	0.30	0.37
FBG	0.88	2.44

**FIGURE 3:**
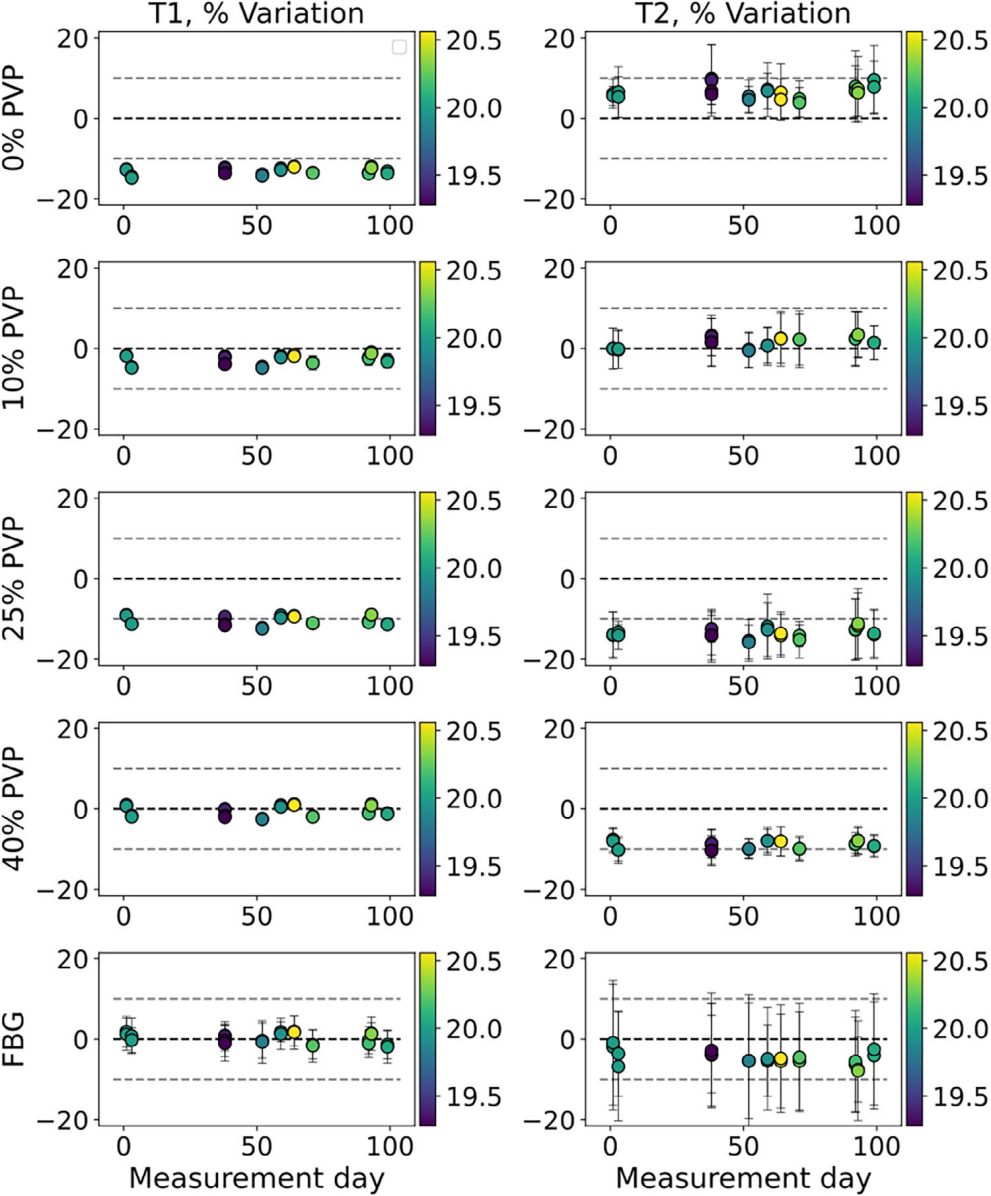
T1 (left) and T2 (right) percent deviation of mean measurement by {day, repeat} compared to the NMR measured value for each material (rows). The scan room temperature is shown in color (colorbar in°C). X‐axes show measurement day, where day 1 is the first day of in vivo acquisition. Coefficients of variation are plotted (error bars), though for some T1 measurements, the error bar is within the marker. The dashed lines show ± 10 % variation from the NMR‐measured value. Over the 99‐day study duration, there was no systematic change in the deviation from the NMR‐measured value.

During the 99‐day study duration, most of the T1 and T2 measurements of the phantom materials were within 10 % variation of the NMR measured value (Figure 3). The 0 % PVP T1 measurement and the T2 measurement of 25 % PVP were outside of 10 % deviation; both were underestimated compared to the NMR measured value. Additionally, all the measurements except for FBG T2 were repeatable with a CV less than or equal to 5 % across the 99 days (Table 3), even if the measurement was not accurate (e.g., 25 % PVP T2). The FBG T2 measurement CV was 11.84 %. During this time, the temperature varied between 19.28°C and 20.56°C, and we did not observe any temperature‐related variation in the T1 nor T2 measurements. Figure 3 shows the temperature for each measurement's variation from the NMR measured value; there is no systematic change observed with changes in temperature.

**TABLE 3:** T1 and T2 coefficient of variation (CV) for each phantom material over the 99‐day study shows coefficient variation of less than or equal to 5.00 % for all measurements except FBG T2, for which the variation was 11.84 %.

Material	T1 CV (%)	T2 CV (%)
0 % PVP	0.95	1.47
10 % PVP	1.74	4.82
25 % PVP	1.35	5.00
40 % PVP	1.46	2.97
FBG	3.51	11.84

### 3.2 In vivo measurements

**FIGURE 4:**
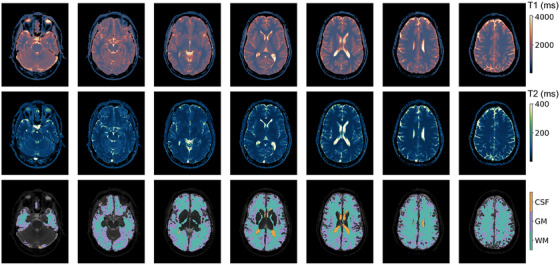
Representative example of in vivo MRF scan results from one participant for T1 (top row) and T2 (middle row). Seven slices were acquired and are displayed inferior to superior from left to right. The bottom row shows segmentation of CSF, GM, and WM for each slice, with a gray‐scale version of the T1 map shown in the background for anatomical reference. CSF, cerebrospinal fluid; GM, gray matter; MRF, magnetic resonance fingerprinting; WM, white matter.

**FIGURE 5:**
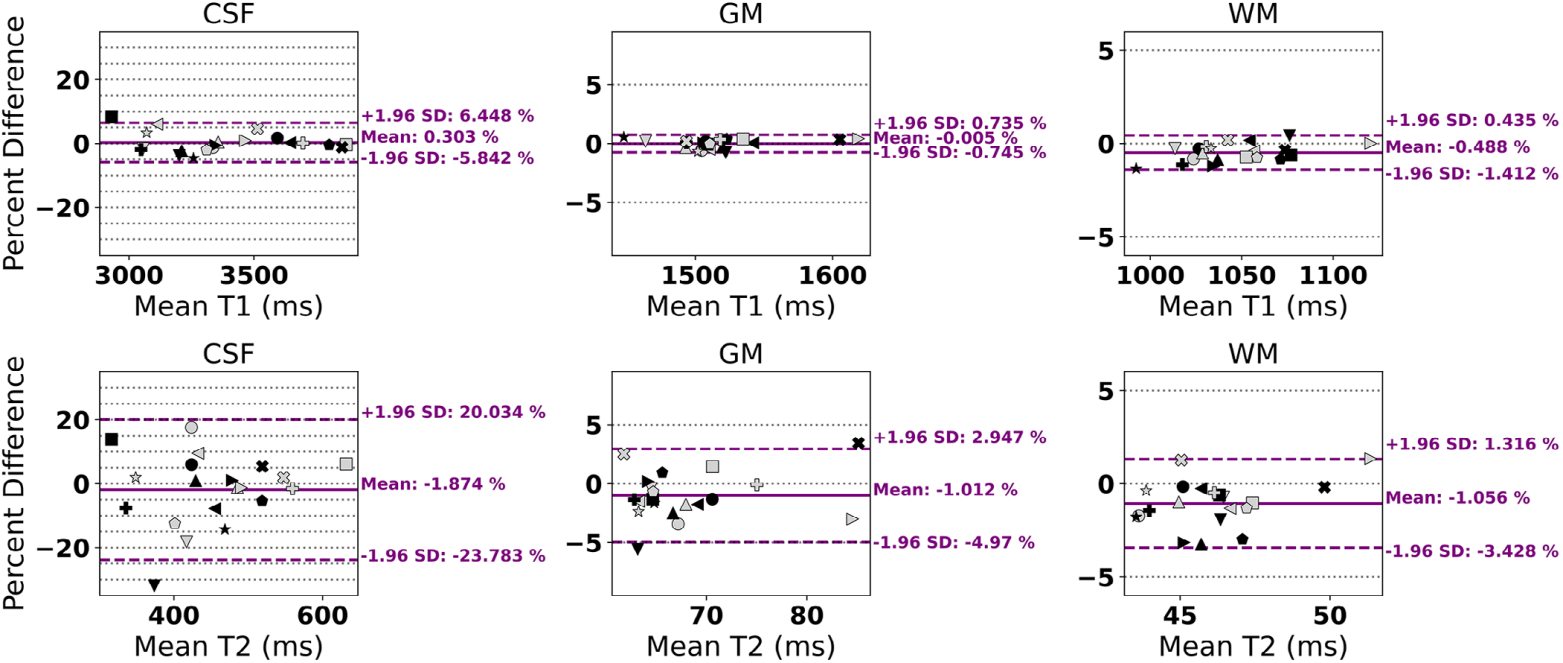
Bland–Altman plots of CSF (left), GM (center), and WM (right) for T1 (top row) and T2 (bottom row) for each participant's test‐retest measurement. Each marker (different color/shape) represents a single participant. Percent differences between each test‐retest measurement are calculated and plotted as a function of the mean test‐retest value. The mean (solid line, purple) and 1.96*standard deviation (dashed lines, purple) were calculated for each measurement type and tissue. Gray horizontal dotted grid lines at 5 % intervals are included to aid in visual clarity. GM, gray matter; MRF, magnetic resonance fingerprinting; WM, white matter.

Figure 4 shows one participant's T1 and T2 measurements along with the segmentation algorithm's output. Bland‐Altman plots of participant test‐retest measurements are shown for each brain region (Figure 5). There is minimal bias between the test‐retest measurements: across all tissue types for T1 and T2, the mean differences are < ± 2.0 %. The variation in CSF measurements was larger, and two participants had variation beyond the confidence interval. Test‐retest variation for CSF, GM, WM was 2.3 %, 0.31 %, and 0.57 % for T1 and 8.3 %, 1.9 %, and 1.3 % for T2.

**FIGURE 6:**
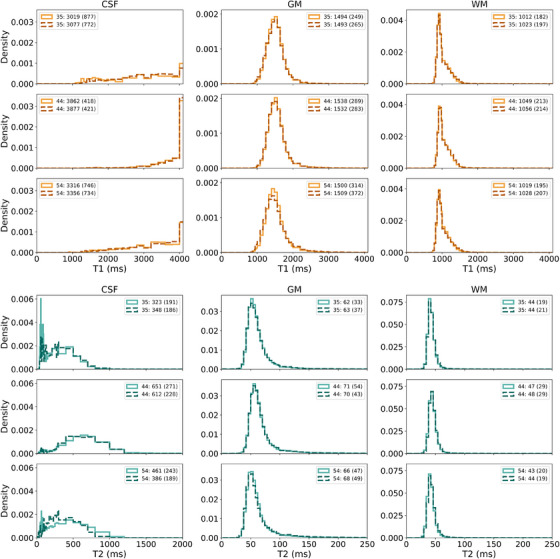
CSF, GM, and WM histograms of T1 (top 3 rows) and T2 (bottom 3 rows) measurements for three representative participants, aged 35, 44, and 54 (arranged from top to bottom). Bin sizes are twice the MRF dictionary step sizes. Histograms of repeat measurements are shown in light or dark orange (T1) or aqua (T2), and the participant's age (years), measurement mean (ms), and measurement standard deviation (ms) are indicated in each legend. To better visualize the T2 histograms, T2 for CSF is plotted to 2000 ms, and T2 for GM and WM is plotted to 250 ms.

**FIGURE 7:**
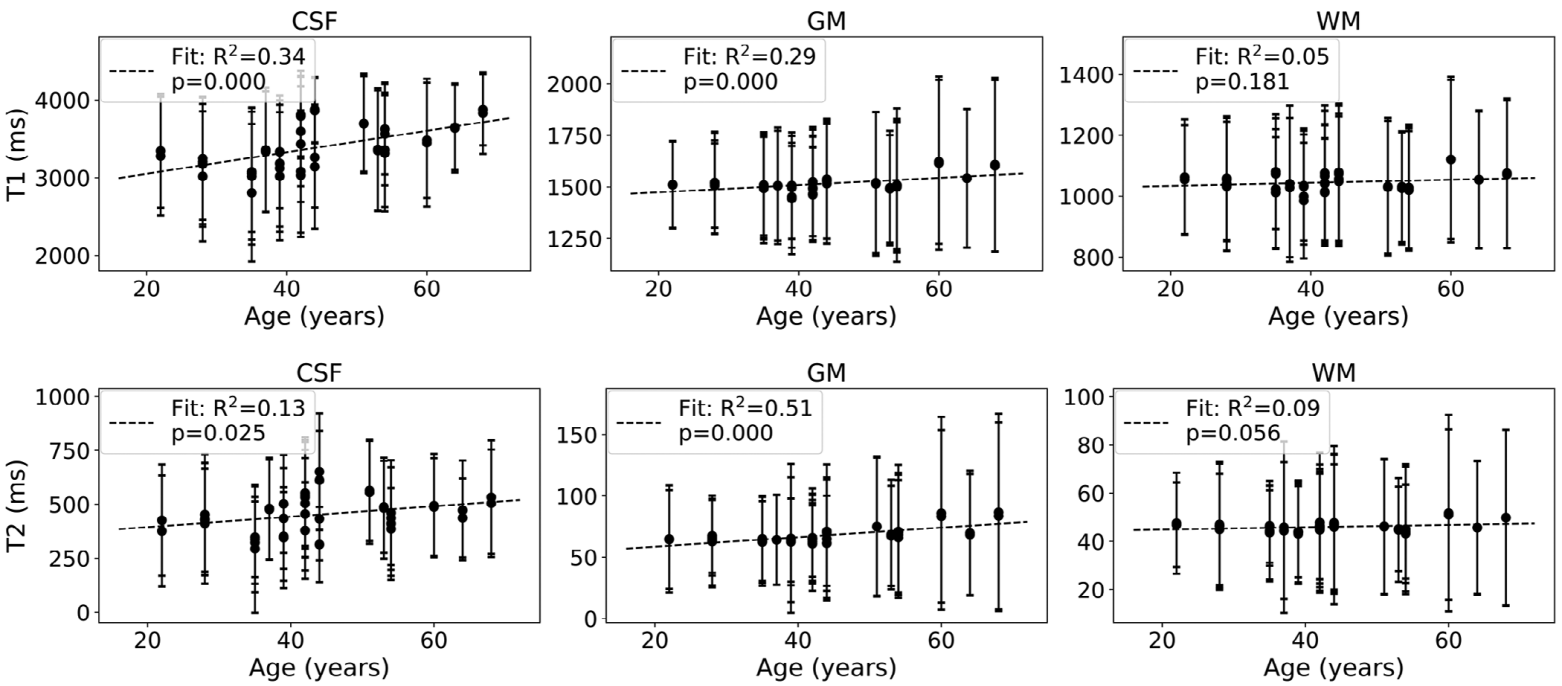
In vivo measurement trends with age. Mean measurement values are plotted with standard deviations (error bars) for T1 (top row) and T2 (bottom row) for CSF, GM, and WM. Linear regressions were performed, and resulting fits are plotted (dashed lines). The R^2^ and *p* values for each fit are indicated. While there is confidence in a relationship between age and relaxation (*p* < 0.05) for all regions except WM, the linear fit is not representative of the relationship (low R^2^ values).

Qualitatively, T1 and T2 histograms of the brain regions are similar for different participant ages, as shown for representative participants aged 35, 44, and 54 years old (Figure 6). Additionally, these histograms illustrate the repeatability of the MRF sequence, particularly in the GM and WM. Age‐related trends in T1 and T2 (Figure 7) indicate that while there is confidence in a relationship between age and relaxation for all regions except WM (*p* < 0.05), the linear fit is not representative of the relationship; the R^2^ values range from 0.1 to 0.5. Table S2 shows results from fitting age‐related trends for each segmented region of the SynthSeg output. There is no relationship between region size and goodness of age‐related fits (high R^2^, low p value).

Mean and standard deviation T1 and T2 were calculated over all participants for each brain region for CSF, GM and WM: 3383 ± 286 ms, 1516 ± 39 ms, 1046 ± 29 ms for T1 and 453 ± 82 ms, 68 ± 7 ms, and 46 ± 2 ms for T2.

Table 4 shows test‐retest variation and volume for each segmented region of the SynthSeg output. The variation tends to increase as the segmented volume falls below 1 cm^3^. Figures S3–S7 show Bland‐Altman plots for the five smallest regions (Inferior Lateral Ventricle, 3rd Ventricle, Accumbens Area, 4th Ventricle, Pallidum).

**TABLE 4:** T1 and T2 test‐retest variation and tissue volume for each segmented tissue. The variation tends to increase as the segmented volume falls below 1 cm^3^.

		Test‐retest variation (%)		Volume (cm^3)	
Datatype	Tissue	Left	Right	Left	Right
T1	Inferior Lateral Ventricle	7.95	13.68	0.03	0.21
	3rd Ventricle	12.82		0.13	
	Accumbens Area	20.58	3.40	0.35	0.28
	4th Ventricle	9.54		0.61	
	Pallidum	4.38	2.48	0.90	1.07
	Amygdala	1.43	1.65	1.25	1.29
	Ventral DC	2.92	2.30	2.15	1.65
	Hippocampus	2.07	1.68	2.15	2.13
	Caudate	3.88	2.78	2.31	2.40
	Putamen	0.73	0.77	3.94	4.01
	Thalamus	2.03	2.21	5.50	5.58
	CSF	2.96	2.29	5.92	6.10
	Cerebellum White Matter	3.51	2.39	6.15	5.27
	Brain‐Stem	1.20		10.48	
	Cerebellum Cortex	1.22	1.67	18.64	16.16
	GM	0.38	0.35	140.05	143.05
	WM	0.64	0.63	195.44	193.27
T2	Inferior Lateral Ventricle	18.27	33.14	0.03	0.21
	3rd Ventricle	19.71		0.13	
	Accumbens Area	54.42	7.15	0.35	0.28
	4th Ventricle	41.81		0.61	
	Pallidum	11.34	7.09	0.90	1.07
	Amygdala	5.12	5.46	1.25	1.29
	Ventral DC	9.57	6.86	2.15	1.65
	Hippocampus	4.91	3.80	2.15	2.13
	Caudate	11.11	8.44	2.31	2.40
	Putamen	2.16	1.88	3.94	4.01
	Thalamus	5.51	8.35	5.50	5.58
	CSF	9.66	9.58	5.92	6.10
	Cerebellum White Matter	5.74	4.52	6.15	5.27
	Brain‐Stem	3.91		10.48	
	Cerebellum Cortex	3.13	3.04	18.64	16.16
	GM	1.69	2.04	140.05	143.05
	WM	1.41	1.46	195.44	193.27

## 4 DISCUSSION

This study examined accuracy, test‐retest repeatability, and long‐term measurement reproducibility of MRF T1 and T2 in a phantom concurrent with in vivo acquisitions. Over the 99‐day study, the phantom measurements were repeatable and reproducible, and no changes were detected in the MRF measurement of T1 and T2 relaxation times on this system. We report phantom‐validated MRF T1 and T2 relaxation times of 20 participants for CSF, GM, and WM and smaller brain regions.

The phantom results indicate that the MRF sequence is repeatable in test‐retest acquisition and reproducible over time. Similarly, previous studies found reproducibility in MRF measurements.^3^, ^7^, ^8^, ^9^ Some measurement variation is expected since this experiment was not temperature‐controlled.^23^ However, the measurement variation did not correlate with the temperature variation, indicating that the measurement variation source was likely noise in the measurement itself rather than variations in temperature. Throughout the study, the measurement variations were minimal in the phantom, which validates the in vivo measurements.

In this phantom, the FBG mimic is the best representation of the T1 and T2 values of GM and WM. However, the FBG mimic T2 measurement also had the most variability. This variation is largely attributed to positional dependence and is also apparent in the T1 measurement (Figure S2). At a single ROI location, the T2 variability is substantially less than across all ROIs (maximum of 3.67 % at one ROI compared to 11.84 % across all ROIs, Table S1). Interestingly, the B1 map did not have the same positional dependence across the ROIs (Figure S2).

In vivo T1 and T2 measurements are comparable to other MRF results at 3 T^7^. The phantom‐validation of this study increases the confidence in these reported numbers. Additionally, Bland‐Altman analysis showed agreement between in vivo test‐retest measurements. As observed here, T2 has previously been observed to have larger variation than T1.^8^ Test‐retest variation was low for additional brain region segmentations (beyond CSF, WM, and GM) for volumes > 1 cm^3^. For smaller volumes, the test‐retest variation increased substantially. This could be caused by inaccurate segmentation in SynthSeg, or by partial volume effects from the 1 mm x 1 mm x 5 mm voxel size. Future work may include image registration to evaluate a pixel‐by‐pixel comparison of the test‐retest data. Note, for CSF in vivo results, the ventricles are expected to have a larger variation due to pulsation artifacts. We observed increased relaxation times with increasing age for most brain regions. Studies at 3 T have shown similar increases in T2 with age for some regions.^24^ At 1.5 T, studies have shown increases in T1 with age for GM and WM.^25^

This study used a research version of the vendor's FDA‐approved MRF implementation for brain imaging. These results are limited by the accuracy of the MRF sequence and dictionary signal model. In most cases, the dictionary step size is the same order of magnitude as the observed population‐level variability. Specifically, the standard deviation of CSF, GM, and WM T1 measurements were 286, 39, and 29 ms, and for T2 measurements, 82, 7, and 2 ms, respectively. The corresponding step sizes for these dictionary ranges were for T1 100, 40, and 20–40 ms (this mean value is close to the change in step size), and for T2 50, 2, and 2 ms. The standard deviation for CSF T1 and T2 measurements are approximately 2–3 times the step size, while the standard deviation for GM and WM T1 and T2 measurements are comparable to the step size. The dictionary step size does impact the performance of the method. Here, we used a research version of the FDA‐approved MRF implementation, and experiments implementing different step sizes were beyond the scope of the current work.

A limitation of this MRF implementation is that it solves the Bloch equations, but does not include magnetization transfer (MT), diffusion or other effects in the signal model, which can contribute measurement error.^26^ In this study, we note the larger discrepancy between the measured MRF and NMR values for the 0 % PVP T1 measurements and the 25 % PVP T2 measurement compared with the other materials. MT effects may be the cause of differences between the NMR and MRF measured T2 values of the higher PVP concentration samples (25 % and 40 % PVP), as samples with higher concentrations of PVP are expected to be more affected by MT. T2 measurement, specifically, is challenging due to its sensitivity to MT and diffusion.^27^, ^28^ The PVP samples do have varying diffusivity, with the higher concentration PVP samples having lower diffusivity. Diffusion effects can result in artificially shorter spin echo T2 values;^29^ however, that effect is opposite to the trend seen in this study. Additionally, complex materials, for example, the FBG material, may exhibit multiple relaxation processes, which are not accounted for in this MRF implementation and may contribute to the higher CV values seen for the FBG material.

The phantom used in this study is primarily a quantitative diffusion phantom and not a relaxometry phantom. However, this work is part of a larger study that required quantitative diffusion measurement. As a result, a single phantom was used for measurement validation. We note, this phantom contains one material (FBG) that has both a T1 and T2 comparable to the measured values in WM and GM. Other phantoms used in previous work (e.g., the ISMRM/NIST system phantom) do not contain any materials that simultaneously represent the T1 and T2 of WM and GM.^30^

One limitation of this study is that participants were imaged at a single time point on a single MRI system. An advantage of the MRF method is its repeatability across different MRI systems, and we did not test that in this study.

We do not know the frequency with which phantom measurements need to be made to validate in vivo measurements. This study provides one data point to begin to answer the clinically relevant question of how often phantom quality assessment must be done in tandem with in vivo measurements. However, the results of this study are limited to this measurement method and this particular MRI system. A study on a different MRI system or on the same MRI system over a different 3 month period might observe issues with the repeatability or reproducibility of the measurement. To address this important question more in vivo studies that incorporate phantom measurements are needed.

In this study, most of the phantom scans were acquired between the adjacent sessions of two participants. Acquiring quantitative phantom data in the time between adjacent participant scans allows contemporaneous phantom data to be acquired with each in vivo dataset, while efficiently using limited scanner time. Additionally, a quantitative phantom data set for each scan day eases the validation of the in vivo data and assessment of scanner longitudinal repeatability.

## 5 CONCLUSION

Phantom measurements characterized the measurement accuracy, repeatability, and reproducibility throughout the 99‐day, 20‐participant study. The MRF T1 and T2 measurements of nominally healthy brain tissues at 3 T are validated through concurrent phantom measurements, and mean values were reported for each tissue.

## AUTHOR CONTRIBUTIONS

Kalina V. Jordanova, Kathryn E. Keenan, and Mathias Nittka conceptualized the study. Mathias Nittka and Guido Buonincontri developed the MRI methods. Kalina V. Jordanova, Kathryn E. Keenan, and Stephen E. Ogier collected and analyzed the MRI data. Stephen E. Russek, Karl F. Stupic, Kathryn E. Keenan, and Cassandra M. Stoffer developed the NMR methods, collected the NMR data, and analyzed these results. Kalina V. Jordanova and Kathryn E. Keenan were responsible for writing the paper. All authors participated in the discussion of results and revisions to the manuscript.

## CONFLICT OF INTEREST STATEMENT

M.N. and G.B. are employees of Siemens Heathineers AG. The other authors have no relevant conflicts of interest to disclose.

## DISCLOSURES

Certain commercial equipment, instruments, software, or materials are identified in this paper in order to specify the experimental procedure adequately. Such identification is not intended to imply recommendation or endorsement by NIST, nor is it intended to imply that the materials or equipment identified are necessarily the best available for the purpose.

## Supporting information



Supporting Information

## Data Availability

The MRF T1 and T2 maps of the phantom and participant measurements in this manuscript are available at: https://doi.org/10.18434/mds2‐3379.

## References

[1] Tofts PS , du Boulay EPGH . Towards quantitative measurements of relaxation times and other parameters in the brain. Neuroradiology. 1990;32(5):407‐415. doi:10.1007/BF00588474 2259435 10.1007/BF00588474

[2] Bojorquez JZ , Bricq S , Acquitter C , Brunotte F , Walker PM , Lalande A . What are normal relaxation times of tissues at 3 T?. Magn Reson Imaging. 2017;35:69‐80. doi:10.1016/j.mri.2016.08.021 27594531 10.1016/j.mri.2016.08.021

[3] Körzdörfer G , Kirsch R , Liu K , et al. Reproducibility and repeatability of MR fingerprinting relaxometry in the human brain. Radiology. 2019;292(2):429‐437. doi:10.1148/radiol.2019182360 31210615 10.1148/radiol.2019182360

[4] Keenan KE , Gimbutas Z , Dienstfrey A , Stupic KF . Assessing effects of scanner upgrades for clinical studies. J Magn Reson Imaging. 2019;50(6):1948‐1954. doi:10.1002/jmri.26785 31111981 10.1002/jmri.26785

[5] Lee Y , Callaghan MF , Acosta‐Cabronero J , Lutti A , Nagy Z . Establishing intra‐ and inter‐vendor reproducibility of T1 relaxation time measurements with 3T MRI. Magn Reson Med. 2019;81(1):454‐465. doi:10.1002/mrm.27421 30159953 10.1002/mrm.27421

[6] Keenan KE , Ainslie M , Barker AJ , et al. Quantitative magnetic resonance imaging phantoms: a review and the need for a system phantom: quantitative MRI Phantoms Review. Magn Reson Med. 2018;79(1):48‐61. doi:10.1002/mrm.26982 29083101 10.1002/mrm.26982

[7] Buonincontri G , Kurzawski JW , Kaggie JD , et al. Three dimensional MRF obtains highly repeatable and reproducible multi‐parametric estimations in the healthy human brain at 1.5T and 3T. NeuroImage. 2021;226:117573. doi:10.1016/j.neuroimage.2020.117573 33221451 10.1016/j.neuroimage.2020.117573

[8] Jiang YMD . Repeatability of magnetic resonance fingerprinting T1 and T2 estimates assessed using the ISMRM/NIST MRI system phantom. Magn Reson Med. 2017;78(4):1452‐1457. doi:10.1002/mrm.26509 27790751 10.1002/mrm.26509 PMC5408299

[9] Dupuis A , Chen Y , Hansen M , et al. Quantifying 3d MR fingerprinting (3d‐mrf) reproducibility across subjects, sessions, and scanners automatically using mni atlases. Magn Reson Med. 2024;91(5):2074‐2088. doi:10.1002/mrm.29983 38192239 10.1002/mrm.29983 PMC10950529

[10] Buonincontri G , Biagi L , Retico A , et al. Multi‐site repeatability and reproducibility of MR fingerprinting of the healthy brain at 1.5 and 3.0 T. NeuroImage. 2019;195:362‐372. doi:10.1016/j.neuroimage.2019.03.047 30923028 10.1016/j.neuroimage.2019.03.047

[11] Jiang Y , Ma D , Seiberlich N , Gulani V , Griswold MA . MR fingerprinting using fast imaging with steady state precession (FISP) with spiral readout. Magn Reson Med. 2015;74(6):1621‐1631. doi:10.1002/mrm.25559 25491018 10.1002/mrm.25559 PMC4461545

[12] Körzdörfer G , Pfeuffer J , Kluge T , et al. Effect of spiral undersampling patterns on FISP MRF parameter maps. Magn Reson Imaging. 2019;62:174‐180. doi:10.1016/j.mri.2019.01.011 30654162 10.1016/j.mri.2019.01.011

[13] Yokota Y , Okada T , Fushimi Y , et al. Acceleration of 2D‐MR fingerprinting by reducing the number of echoes with increased in‐plane resolution: a volunteer study. Magn Reson Mater Phys Biol Med. 2020;33(6):783‐791. doi:10.1007/s10334‐020‐00842‐8 10.1007/s10334-020-00842-8 PMC7669790 32248322

[14] Tan H , Meyer CH . Estimation of *k* ‐space trajectories in spiral MRI. Magn Reson Med. 2009;61(6):1396‐1404. doi:10.1002/mrm.21813 19353671 10.1002/mrm.21813 PMC3256576

[15] Chung S , Kim D , Breton E , Axel L . Rapid *B* _1_ ^+^ mapping using a preconditioning RF pulse with TurboFLASH readout. Magn Reson Med. 2010;64(2):439‐446. doi:10.1002/mrm.22423 20665788 10.1002/mrm.22423 PMC2929762

[16] Muja M , Lowe D . Fast approximate nearest neighbors with automatic algorithm configuration In: *Proceedings of the Fourth International Conference on Computer Vision Theory and Applications*. 2009;1:331‐340.

[17] McGivney DF , Pierre E , Ma D , et al. SVD compression for magnetic resonance fingerprinting in the time domain. IEEE Trans Med Imaging. 2014;33(12):2311‐2322. doi:10.1109/TMI.2014.2337321 25029380 10.1109/TMI.2014.2337321 PMC4753055

[18] Keenan KE , Wilmes LJ , Aliu SO , et al. Design of a breast phantom for quantitative MRI. J Magn Reson Imaging. 2016;44(3):610‐619. doi:10.1002/jmri.25214 26949897 10.1002/jmri.25214 PMC4983524

[19] Boss MA , Dienstfrey AM , Gimbutas Z , et al. Magnetic Resonance Imaging Biomarker Calibration Service: Proton Spin Relaxation Times. National Institute of Standards and Technology; 2018.

[20] Billot B , Greve DN , Puonti O , et al. SynthSeg: segmentation of brain MRI scans of any contrast and resolution without retraining. Med Image Anal. 2023;86:102789. doi:10.1016/j.media.2023.102789 36857946 10.1016/j.media.2023.102789 PMC10154424

[21] Billot B , Magdamo C , Cheng Y , Arnold SE , Das S , Iglesias JE . Robust machine learning segmentation for large‐scale analysis of heterogeneous clinical brain MRI datasets. Proc Natl Acad Sci. 2023;120(9):e2216399120. doi:10.1073/pnas.2216399120 36802420 10.1073/pnas.2216399120 PMC9992854

[22] Fuderer M , Wichtmann B , Crameri F , et al. Color‐map recommendation for mr relaxometry maps. Magn Reson Med. 2025;93(2):490‐506. doi:10.1002/mrm.30290 39415361 10.1002/mrm.30290 PMC11604837

[23] Statton BK , Smith J , Finnegan ME , Koerzdoerfer G , Quest RA . Grech‐Sollars M. Temperature dependence, accuracy, and repeatability of T _1_ and T _2_ relaxation times for the ISMRM/NIST system phantom measured using MR fingerprinting. Magn Reson Med. 2022;87(3):1446‐1460. doi:10.1002/mrm.29065 34752644 10.1002/mrm.29065

[24] Kumar R , Delshad S , Woo MA , Macey PM , Harper RM . Age‐related regional brain T2‐relaxation changes in healthy adults. J Magn Reson Imaging. 2012;35(2):300‐308. doi:10.1002/jmri.22831 21987489 10.1002/jmri.22831

[25] Steen RG , Gronemeyer SA , Taylor JS . Age‐related changes in proton T1 values of normal human brain. J Magn Reson Imaging. 1995;5(1):43‐48. doi:10.1002/jmri.1880050111 7696808 10.1002/jmri.1880050111

[26] Hilbert T , Xia D , Block KT , et al. Magnetization transfer in magnetic resonance fingerprinting. Magn Reson Med. 2020;84(1):128‐141. doi:10.1002/mrm.28096 31762101 10.1002/mrm.28096 PMC7083689

[27] Majumdar S , Orphanoudakis SC , Gmitro A , O'Donnell M , Gore JC . Errors in the measurements of *T* _2_ using multiple‐echo MRI techniques. I. Effects of radiofrequency pulse imperfections. Magn Reson Med. 1986;3(3):397‐417. doi:10.1002/mrm.1910030305 3724419 10.1002/mrm.1910030305

[28] Lebel RM , Wilman AH . Transverse relaxometry with stimulated echo compensation. Magn Reson Med. 2010;64(4):1005‐1014. doi:10.1002/mrm.22487 20564587 10.1002/mrm.22487

[29] Michaeli S , Garwood M , Zhu X , et al. Proton *T* _2_ relaxation study of water, N‐acetylaspartate, and creatine in human brain using Hahn and Carr‐Purcell spin echoes at 4T and 7T. Magn Reson Med. 2002;47(4):629‐633. doi:10.1002/mrm.10135 11948722 10.1002/mrm.10135

[30] Stupic KF , Ainslie M , Boss MA , et al. A standard system phantom for magnetic resonance imaging. Magn Reson Med. 2021;86(3):1194‐1211. doi:10.1002/mrm.28779 33847012 10.1002/mrm.28779 PMC8252537

