# Supplementary material for: Adult brain T1 and T2 values measured at 3 T using magnetic resonance fingerprinting with phantom validation
Source: J Appl Clin Med Phys. 2025 Dec 15;26(12):e70289. doi: 10.1002/acm2.70289 (PMC12703810; doi:10.1002/acm2.70289)
Supplement: Supplementary file 1 — Supporting Information [file ACM2-26-e70289-s001.docx]

### SUPPLEMENTAL MATERIAL

**
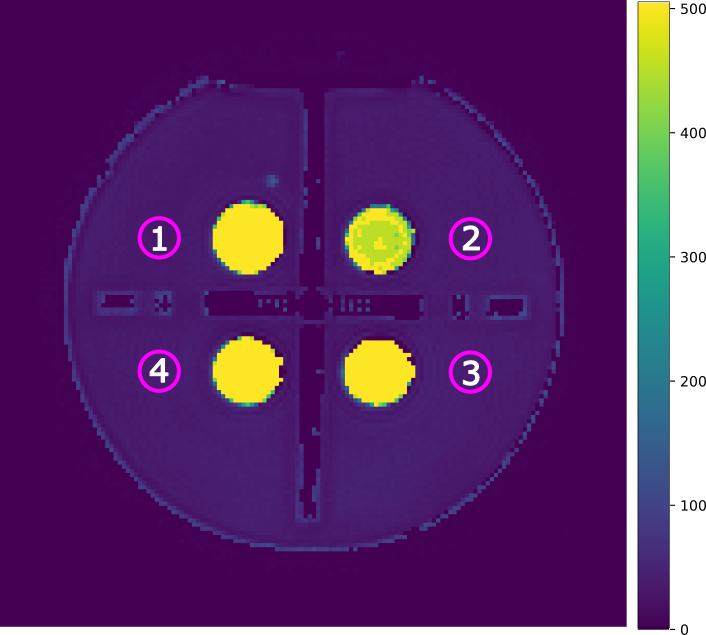
**

**Figure S1:** T2 map of the phantom illustrating the location of the four FBG mimic ROIs.

**
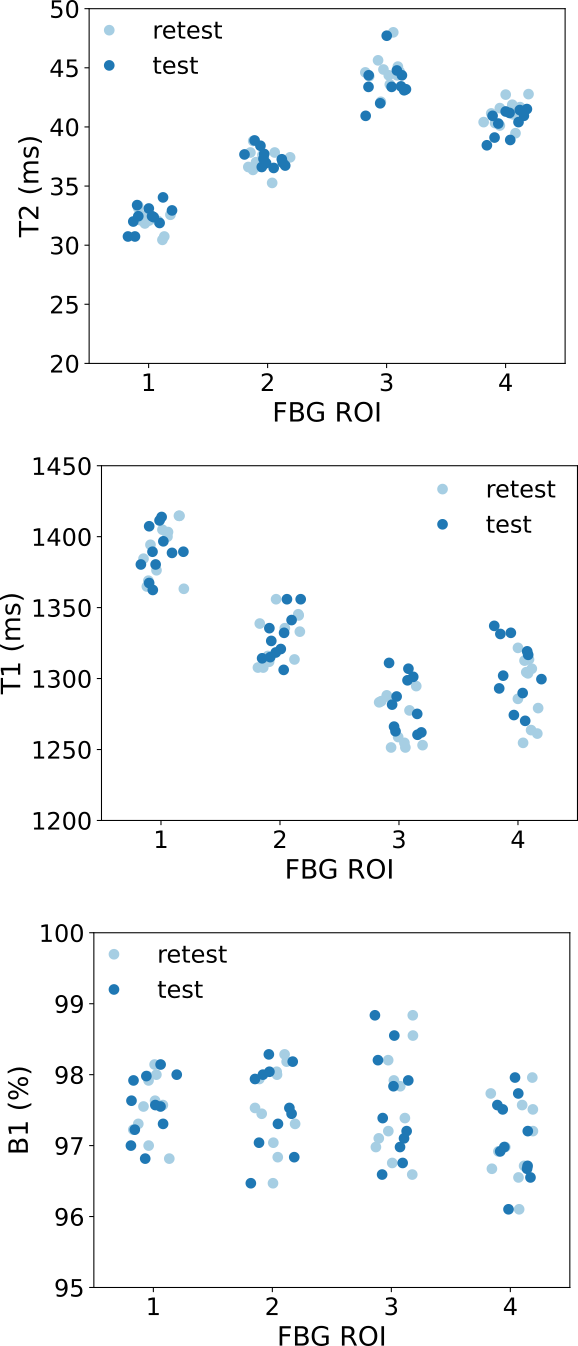
**

**Figure S2:** T2 (ms), T1 (ms), and B1 (%) measurements at the four FBG ROI locations. Results are shown for both the test and retest measurement.


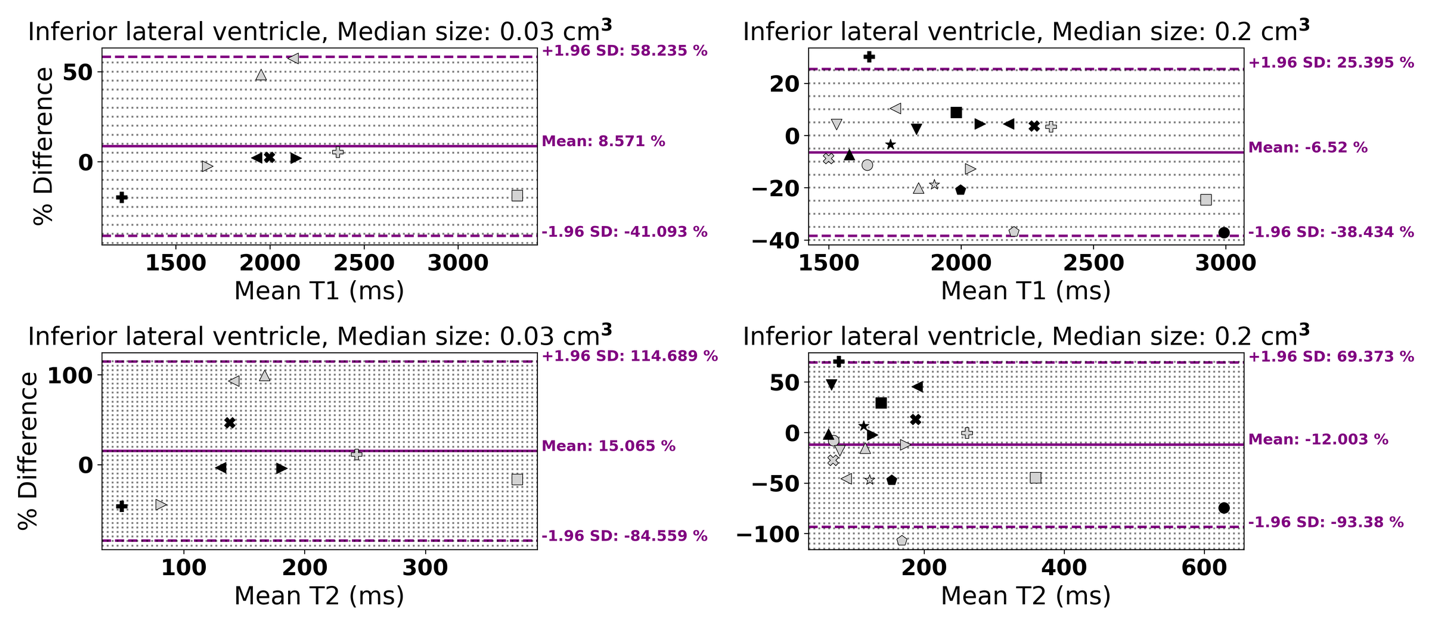


**Figure S3**: Bland-Altman plots of Inferior lateral ventricle for left (left), and right (right) hemisphere segmentations for T1 (top row) and T2 (bottom row), for each participant’s test-retest measurement. Each marker (different color/shape) represents a single participant. Percent differences between each test-retest measurement are calculated and plotted as a function of the mean test-retest value. The mean (solid line, purple) and 1.96*standard deviation (dashed lines, purple) were calculated for each measurement type and tissue. Gray horizontal dotted grid lines at 5 % intervals are included to aid in visual clarity.


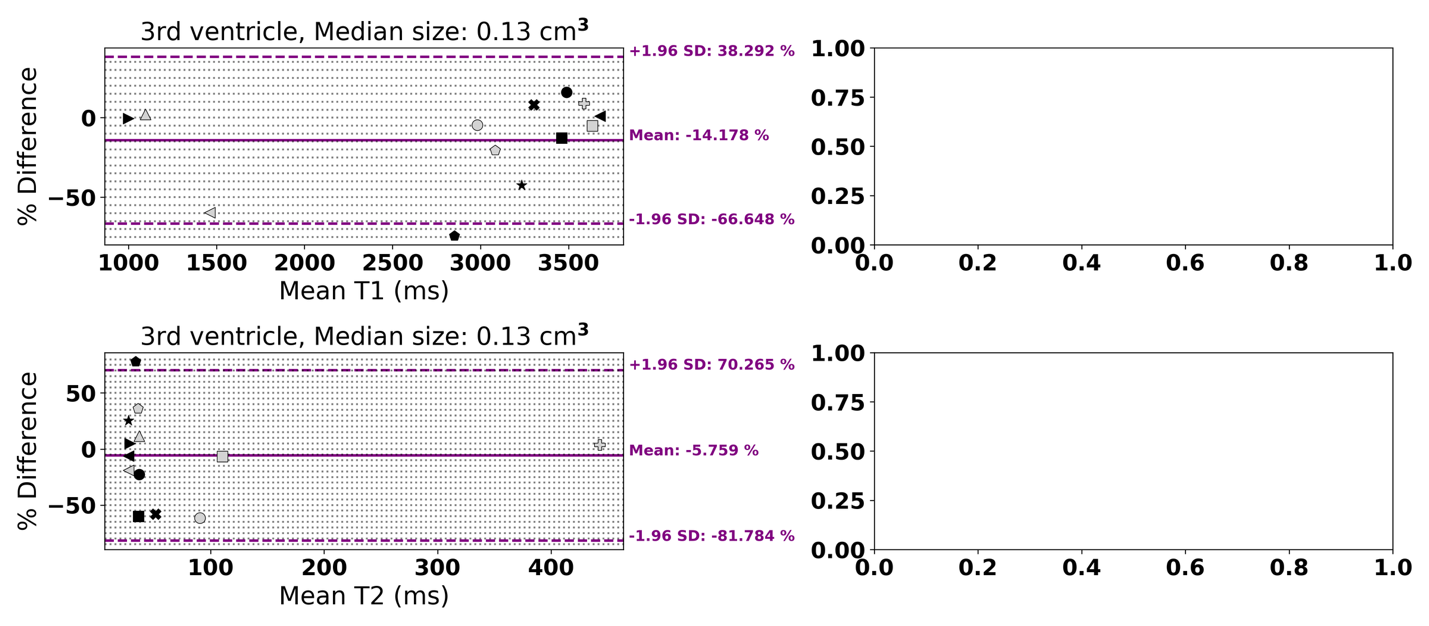


**Figure S4**: Bland-Altman plots of 3^rd^ ventricle segmentations for T1 (top row) and T2 (bottom row), for each participant’s test-retest measurement. Each marker (different color/shape) represents a single participant. Percent differences between each test-retest measurement are calculated and plotted as a function of the mean test-retest value. The mean (solid line, purple) and 1.96*standard deviation (dashed lines, purple) were calculated for each measurement type and tissue. Gray horizontal dotted grid lines at 5 % intervals are included to aid in visual clarity.


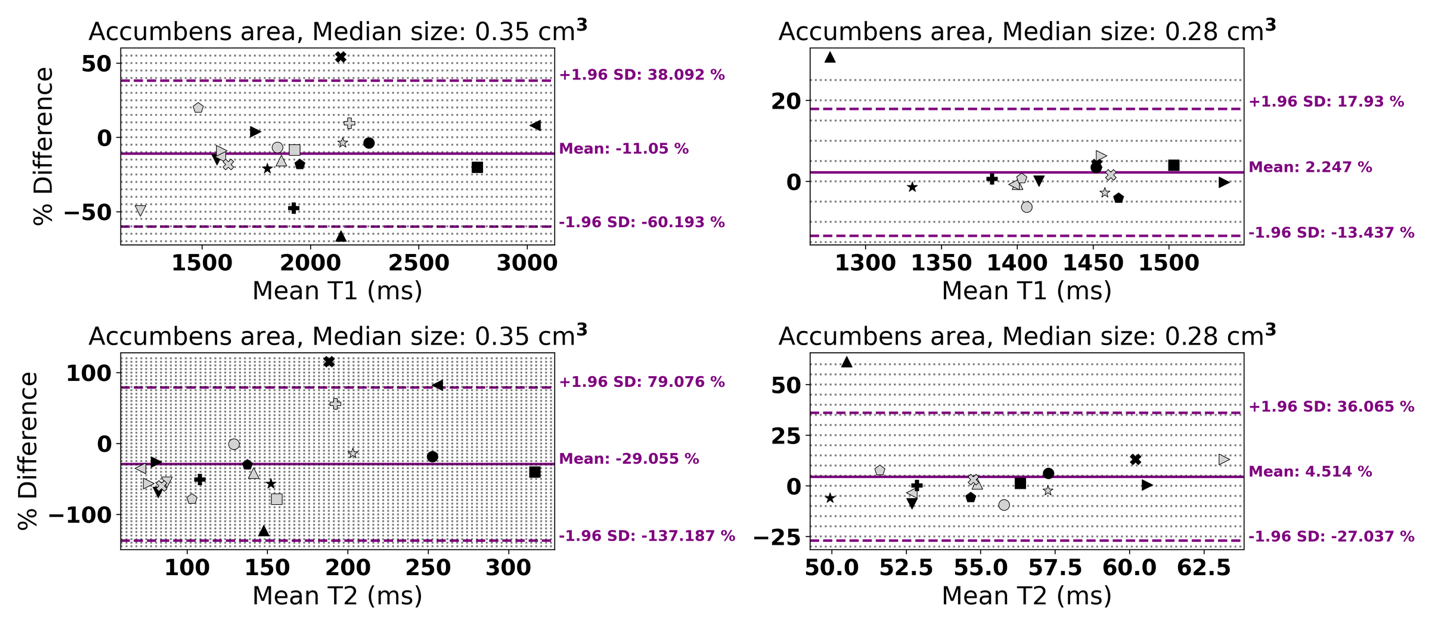


**Figure S5**: Bland-Altman plots of Accumbens area for left (left), and right (right) hemisphere segmentations for T1 (top row) and T2 (bottom row), for each participant’s test-retest measurement. Each marker (different color/shape) represents a single participant. Percent differences between each test-retest measurement are calculated and plotted as a function of the mean test-retest value. The mean (solid line, purple) and 1.96*standard deviation (dashed lines, purple) were calculated for each measurement type and tissue. Gray horizontal dotted grid lines at 5 % intervals are included to aid in visual clarity.


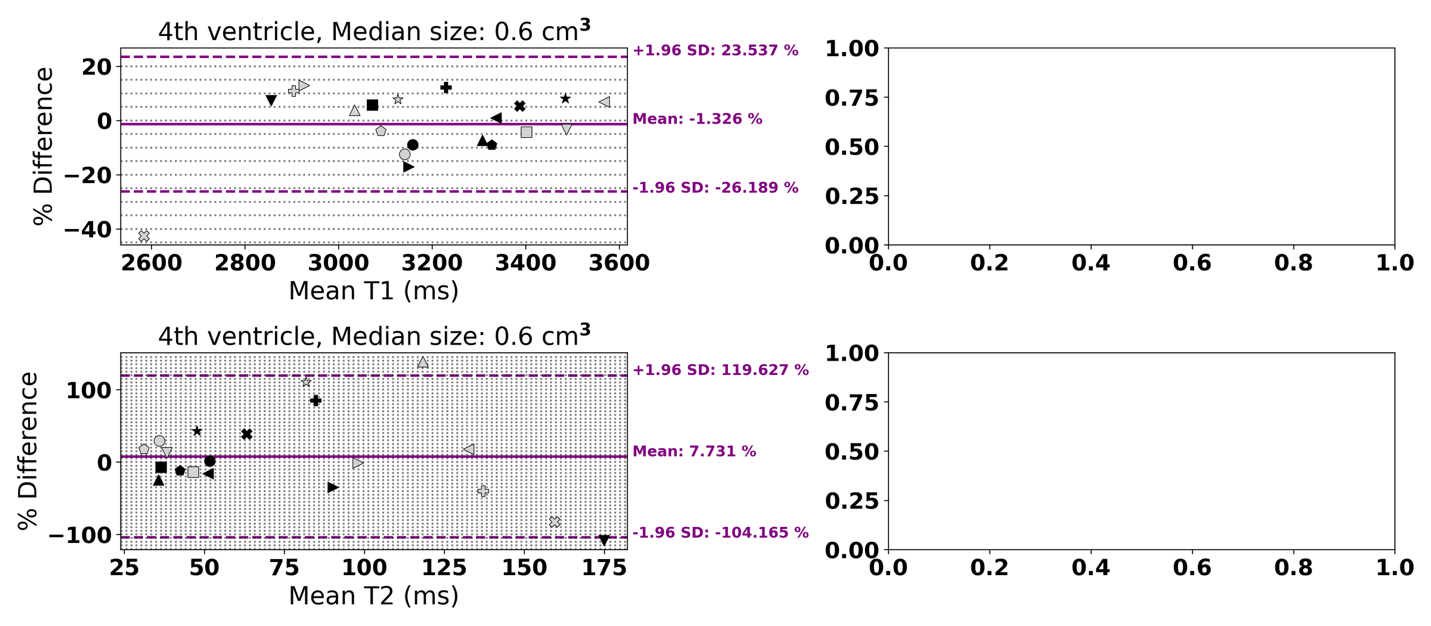


**Figure S6**: Bland-Altman plots of 4^th^ ventricle segmentations for T1 (top row) and T2 (bottom row), for each participant’s test-retest measurement. Each marker (different color/shape) represents a single participant. Percent differences between each test-retest measurement are calculated and plotted as a function of the mean test-retest value. The mean (solid line, purple) and 1.96*standard deviation (dashed lines, purple) were calculated for each measurement type and tissue. Gray horizontal dotted grid lines at 5 % intervals are included to aid in visual clarity.


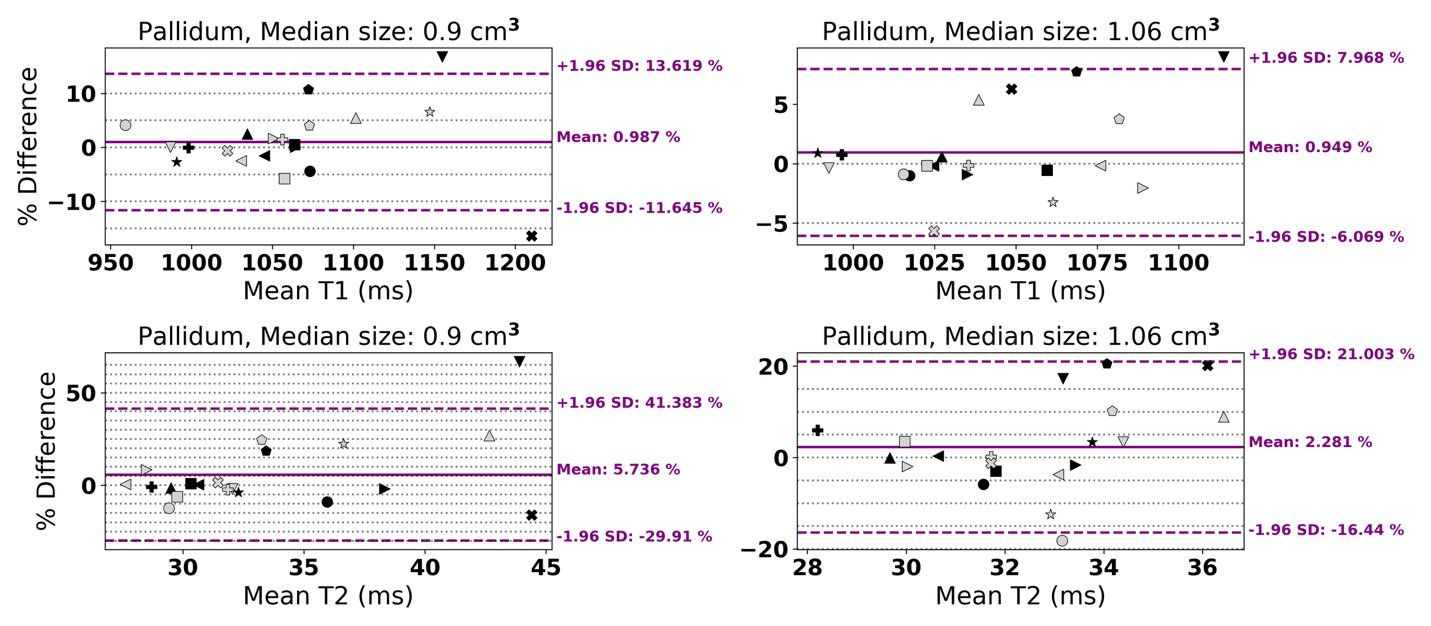


**Figure S7**: Bland-Altman plots of Pallidum for left (left), and right (right) hemisphere segmentations for T1 (top row) and T2 (bottom row), for each participant’s test-retest measurement. Each marker (different color/shape) represents a single participant. Percent differences between each test-retest measurement are calculated and plotted as a function of the mean test-retest value. The mean (solid line, purple) and 1.96*standard deviation (dashed lines, purple) were calculated for each measurement type and tissue. Gray horizontal dotted grid lines at 5 % intervals are included to aid in visual clarity.

| **ROI** | **T1 CV (%)** | **T2 CV (%)** |
| --- | --- | --- |
| 1 | 1.27 | 2.92 |
| 2 | 1.25 | 2.23 |
| 3 | 1.47 | 3.67 |
| 4 | 1.85 | 2.77 |
| Across all ROI | 3.51 | 11.84 |

**Table S1:** T1 and T2 coefficient of variation (CV) for each FBG ROI over the 99-day study shows coefficients of variation of less than 4.00 % for each ROI location, substantially lower than the coefficient of variation across all ROI locations.

| **Datatype** | **Tissue** | **R^2^** | **p value** | **Average volume (cm^2^)** |
| --- | --- | --- | --- | --- |
| T1 | Brain-Stem | 0.00 | 0.871 | 10.5 |
|  | Caudate | 0.00 | 0.752 | 2.4 |
|  | 4th Ventricle | 0.01 | 0.587 | 0.6 |
|  | Hippocampus | 0.01 | 0.504 | 2.1 |
|  | Thalamus | 0.01 | 0.453 | 5.5 |
|  | Amygdala | 0.02 | 0.415 | 1.3 |
|  | Ventral DC | 0.02 | 0.340 | 1.9 |
|  | Putamen | 0.02 | 0.334 | 4.0 |
|  | Pallidum | 0.03 | 0.319 | 1.0 |
|  | WM | 0.05 | 0.181 | 194.4 |
|  | Inferior Lateral Ventricle | 0.05 | 0.157 | 0.1 |
|  | 3rd Ventricle | 0.08 | 0.114 | 0.1 |
|  | Accumbens Area | 0.13 | 0.023 | 0.3 |
|  | GM | 0.29 | 0.000 | 141.6 |
|  | Cerebellum White Matter | 0.33 | 0.000 | 5.7 |
|  | CSF | 0.34 | 0.000 | 6.0 |
|  | Cerebellum Cortex | 0.59 | 0.000 | 17.4 |
| T2 | Ventral DC | 0.00 | 0.824 | 1.9 |
|  | 4th Ventricle | 0.00 | 0.746 | 0.6 |
|  | Putamen | 0.00 | 0.698 | 4.0 |
|  | Caudate | 0.01 | 0.618 | 2.4 |
|  | Amygdala | 0.01 | 0.529 | 1.3 |
|  | 3rd Ventricle | 0.03 | 0.384 | 0.1 |
|  | Pallidum | 0.05 | 0.170 | 1.0 |
|  | Inferior Lateral Ventricle | 0.07 | 0.105 | 0.1 |
|  | WM | 0.09 | 0.056 | 194.4 |
|  | Hippocampus | 0.09 | 0.053 | 2.1 |
|  | Accumbens Area | 0.11 | 0.036 | 0.3 |
|  | CSF | 0.13 | 0.025 | 6.0 |
|  | Thalamus | 0.15 | 0.015 | 5.5 |
|  | Brain-Stem | 0.22 | 0.002 | 10.5 |
|  | Cerebellum White Matter | 0.45 | 0.000 | 5.7 |
|  | Cerebellum Cortex | 0.47 | 0.000 | 17.4 |
|  | GM | 0.51 | 0.000 | 141.6 |

**Table S2**: R2, p values, and average volume for T1 and T2 measurement trends with respect to age, for each tissue segmented using SynthSeg.
